# Public Opinion Polarization by Individual Revenue from the Social Preference Theory

**DOI:** 10.3390/ijerph17030946

**Published:** 2020-02-04

**Authors:** Tinggui Chen, Qianqian Li, Peihua Fu, Jianjun Yang, Chonghuan Xu, Guodong Cong, Gongfa Li

**Affiliations:** 1 School of Statistics and Mathematics, Zhejiang Gongshang University, Hangzhou 310018, China; 2 School of Management and E-Business, Zhejiang Gongshang University, Hangzhou 310018, China; lzsfamy@yeah.net (Q.L.); fph@mail.zjgsu.edu.cn (P.F.); 3 Department of Computer Science and Information Systems, University of North Georgia, Oakwood, GA 30566, USA; Jianjun.Yang@ung.edu; 4 School of Business Administration, Zhejiang Gongshang University, Hangzhou 310018, China; talentxch@zjgsu.edu.cn; 5 School of Tourism and Urban-Rural Planning, Zhejiang Gongshang University, Hangzhou 310018, China; cgd@mail.zjgsu.edu.cn; 6 Hubei Key Laboratory of Mechanical Transmission and Manufacturing Engineering, Wuhan University of Science and Technology, Wuhan 430081, China; ligongfa@wust.edu.cn

**Keywords:** public opinion polarization, social preference, individual interaction, individual revenue

## Abstract

Social conflicts occur frequently during the social transition period and the polarization of public opinion happens occasionally. By introducing the social preference theory, the target of this paper is to reveal the micro-interaction mechanism of public opinion polarization. Firstly, we divide the social preferences of Internet users (network nodes) into three categories: egoistic, altruistic, and fair preferences, and adopt the revenue function to define the benefits obtained by individuals with different preferences among their interaction process so as to analyze their decision-making behaviors driven by the revenue. Secondly, the revenue function is used to judge the exit rules of nodes in a network, and then a dynamic network of spreading public opinion with the node (individual) exit mechanism is built based on a BA scale-free network. Subsequently, the influences of different social preferences, as well as individual revenue on the effect of public opinion polarization, are analyzed through simulation experiments. The simulation results show that (1) Different social preferences demonstrate different influences on the evolution of public opinions, (2) Individuals tend to interact with ones with different preferences, (3) The network with a single preference or a high aggregation is more likely to form public opinion polarization. Finally, the practicability and effectiveness of the proposed model are verified by a real case.

## 1. Introduction

When hot events break out on the Internet, network forums and discussion groups related to the topic will be generated along with the propagation of these hot events. When people communicate in forums or discussion groups, fierce disputes or mutual affirmation of different views will lead to the popularity of public opinion and generate one or more extreme attitude values, which are called public opinion polarization. Generally speaking, the effective usage of public opinion polarization can improve the public’s trust in government, and also increase customer loyalty to enterprises [1]. For example, in the Spring Festival of 2020, when China’s novel coronavirus broke out, the Henan provincial government acted decisively and responded effectively. Soon afterwards, topics such as “How to Steal the Governor of Henan Province” and “Henan Province hand over the full score answer sheet” appeared in the network to occupy the top of the hot search list, which greatly enhanced the credibility of the Henan provincial government. However, since every coin has two sides, the phenomenon of public opinion polarization also easily results in network violence and even extends such behavior from online to offline, such as sit-downs, protest marches, and other social group behaviors that are not conducive to social stability. As a result, research on the emergence mechanism of public opinion polarization is of important theoretical and practical significance.

At present, there are relatively few studies on the public opinion polarization, and most of them mainly adopt methods such as macro statistics [2] or mathematical modeling [3,4] to analyze the main characteristics of the formation and propagation of polarization phenomena at the macro level. However, these methods generally lack studies on the interaction mechanism of micro individuals. In fact, only by fully exploring the internal driving force for individuals to participate in the interaction of views in the network, as well as analyzing the direct reasons behind their decision-making behaviors, can the internal mechanism of polarization phenomenon be further understood. In the process of micro-individual interactions, every individual is different, which makes him/her have different social preferences. These differentiated social preferences will bring different revenues for individuals to participate in the interactions, thus prompting them to take different interactive behaviors driven by revenues, and ultimately affecting public opinion polarization on the whole network. Currently, the social preference theory [5,6] has widely applied to individual behavioral decisions in the field of social and economic activities but is rarely used in the field of public opinion evolution. In this paper, we introduce the social preference theory and build a public opinion polarization model for analyzing individual interaction behavior from the perspective of gain for different individuals. As far as we know, it is the first time that the social preference theory has been adopted for public opinion polarization. Subsequently, the effects of different social preferences, network structure, and individual revenues on public opinion polarization are explored by simulation experiments and some observations are obtained through simulation experiments. Finally, the practicability and effectiveness of the proposed model are verified by a real case.

The structure of this paper is organized as follows: Section 2 is a literature review and points out the innovations of this paper. Section 3 constructs the revenue model of individual interaction with different social preferences. Section 4 discusses the influences of various parameters on opinion polarization by simulation experiments. Subsequently, a real case is used to testify the practicability and effectiveness of our proposed model in Section 5. Section 6 is the conclusions and prospects of work in the future.

## 2. Literature Review

For research on group polarization, Professor Sunstein [1] proposed in 2002 that if there was some bias in the opinions of group members at the beginning, such bias would be strengthened after discussion, and finally, an extreme opinion would be formed, which is the public opinion polarization. Subsequently, many scholars have also studied the phenomenon of public opinion polarization and have made some achievements. For example, Liu et al. [7] studied the overconfidence problem in viewpoint interactions among different individuals in 2019 and proposed that overconfidence would have a negative impact on large-scale group decision-making and reduce the consistency of decision-making. In 2017, Dong et al. [8] explored the issue of consensus under the guidance of multiple leaders and found that the dynamic change process of leaders could promote public opinion polarization. Dandekar et al. [9] proposed in 2013 that if individuals in the network had enough prejudice at the initial moment, such prejudice would lead to public opinion polarization after a lot of interactions. Etesami and Basar [10] studied the public opinion polarization phenomenon in high dimensions in 2014 and found that whether it forms single polarization, bi-polarization, or multi-polarization depended on the openness of the mind. In 2013, Li et al. [11] discussed the internal characteristics of the gradual shift of public opinion from bi-polarization to single polarization by analyzing its convergence characteristics. Leon-Medini [12] established a new multi-agent model to study the final trend of public opinion evolution in 2019. In 2017, by comparing networks with different structures, Li and Xiao [13] found that the increase of average network would raise the polarization degree, and the viewpoints of different dimensions would affect each other in the network evolution, thus reducing the polarization phenomenon. In 2019, Chen et al. [14] considered the influence of heterogeneous individuals in the network on the public opinion polarization, and also introduced the concept of dynamic conformity to discuss the influence of “silent spiral” phenomenon on the polarization of public opinion. However, the driving force for individuals to participate in the interaction was not considered in the above paper.

All in all, the literature mentioned above is focused on the analysis of the general propagation rule of opinion polarization phenomenon at the macro level, or the simulation of the evolution process of the entire public opinion polarization by establishing the model of public opinion propagation so as to explore the complex behaviors of the macro system as a whole. However, studies are rarely related to individual interaction mechanisms at the micro level. Due to the complexity of netizens, there are many influence factors involved in their interactions, so quantitative analysis is difficult to deal with in this issue. Considering that every individual is driven by revenue during interactions, individuals are only willing to engage in perspective interactions when they can obtain certain benefits. Individuals with different social preferences have diverse benefits from each interaction. Based on this, the benefits brought by different preferences can explain the essential motivations of the different behavior choices of individuals in public opinion communication. Therefore, it is of great practical significance to use the social preference theory to study the public opinion polarization phenomenon so as to better explain the interrelationships among various factors that affect the public opinion polarization phenomenon.

There are also many existing studies on the social interaction, social conflict, and social impact of public opinion analysis. For example, in 2019, Zhou [15] used a structural equation model to explore the impact of social interaction on users’ social and commercial willingness. In 2019, Kim et al. [16] studied the relationship between offline social interaction and online shopping. In 2016, Szczecinska-Musielak [17] discussed the usefulness of social conflict theory as a theoretical framework for analyzing the conflict in Northern Ireland. In 2018, Cuppen [18] emphasized the value of social conflict in energy policy and planning and discussed several basic characteristics of social conflict. Zhou [19] proposed in 2019 that social support, including information support and emotional support, had a significant impact on social influence. Peng et al. [20] presented an evaluation model to measure both direct and indirect influence based on the social relationship graph, by introducing friend entropy and interaction frequency entropy to describe the complexity and uncertainty of social influence. All the above literature study network public opinion from macro perspectives and mainly use qualitative methods to analyze opinion evolution. However, micro individual interactions can determine the macro emergence of group behavior. As a result, it is necessary to explore public opinion polarization from micro individual interactions. That is why we introduce social preference theory to measure individual revenues in interactions.

In addition, some scholars have attempted to explain the evolution mechanism of public opinion through the preference theory. For instance, in 2015, Alizadeh et al. [21] investigated the interplay of homophily, rejection, and in-group cooperation drivers on the formation of opinion clusters and the emergence of extremist, radical opinions. Their model was to explicitly explore the effect of in-group favoritism on the macro-level, collective behavior of opinions. In 2019, Banisch and Olbrich [22] analytically characterized sufficient conditions for the stability of bi-polarization with the consideration of the social feedback process. Comparing with previous models, their model highlighted an effective experience-based route to polarization. In 2019, Dong et al. [23] believed that while interacting with their views, interlocutors often hid their real preferences and expressed different preferences to different persons. Furthermore, by dividing the individual preferences into real preference, communication preference, and public preference, they studied the deceptive interaction and the preference evolution of heterogeneous trust in the network. In 2018, Navarro-Martinez et al. [24] incorporated the expected utility theory to build a model of the dynamic preference where the preferences selected by the decision-makers for each interaction were recorded and compared with the previous preferences. In 2018, Guevara et al. [25] measured the dynamic change of individual preference values during interaction by establishing a continuous opinion space. In 2010 and 2014, Cabrerizo et al. [26,27] described a concept of a granular fuzzy preference relation and gave the influence of individual preference on decision results in a fuzzy group. In 2007, Herrera-Viedma et al. [28] studied the impact of information shortage for incomplete fuzzy preference relations. Pérez et al. [29] presented a novel model in 2016 that gathered the experts’ initial opinions and provided a framework to represent the influence of a given expert over the other(s). With this proposal, it was feasible to estimate both the evolution of the group decision-making process and the final solution before carrying out the group discussion process and consequently foreseeing possible actions. In 2018, Gayle and Khorunzhina [30] put forward an extended model based on fuzzy consensus while evaluating resources. By this model, rational decisions could be made so as to reduce the impact of individual preference on evaluation accuracy. Barseghyan et al. [31] studied the family risk preferences through expectation utility theory in 2016. All these studies mentioned above are of great significance to the field of public opinion, but they mainly focus on qualitative analysis and lack quantitative modeling and analysis process, leading to shortages of intuitiveness and validity of the characterization of complex and changeable public opinion evolution.

To sum up, current research on the public opinion polarization mostly analyzes the causes of polarization by simulating the macro rule of the public opinion evolution, but rarely conducts more in-depth studies on the benefits of individual interaction and the social preferences held by individuals at the micro level. Actually, the interaction of micro individuals determines the emergence of macro groups. At present, studies on the applications of social preference theory to the phenomenon of public opinion polarization are still rare. During the public opinion evolution, individuals with different preferences will get different benefits, which will drive them to make different decisions while interacting with each other. However, the diverse interaction behaviors of these individuals ultimately lead to different public opinion polarization effects. In addition, in terms of individual interaction, the intimacy and friendliness of the participants must be considered. In general, individuals tend to interact with ones with similar views or close friendships, and their interaction yields will be higher. Therefore, this paper firstly introduces the concept of the intimacy as well as friendliness and then establishes individual revenue function. Subsequently, a new public opinion polarization model to explore opinion propagation rule is set up. In addition, according to the individual revenue function, a network structure considering node exit mechanism is built on the basis of a BA scale-free network [32], which expands the static network in previous studies [33,34] into the dynamic one, making it more realistic. Finally, the influences of different social preferences and individual revenue functions on the public opinion polarization effects are analyzed through simulation experiments, and a real case is also given to verify the practicability and effectiveness of the proposed model.

## 3. Model Construction

**Figure 1:**
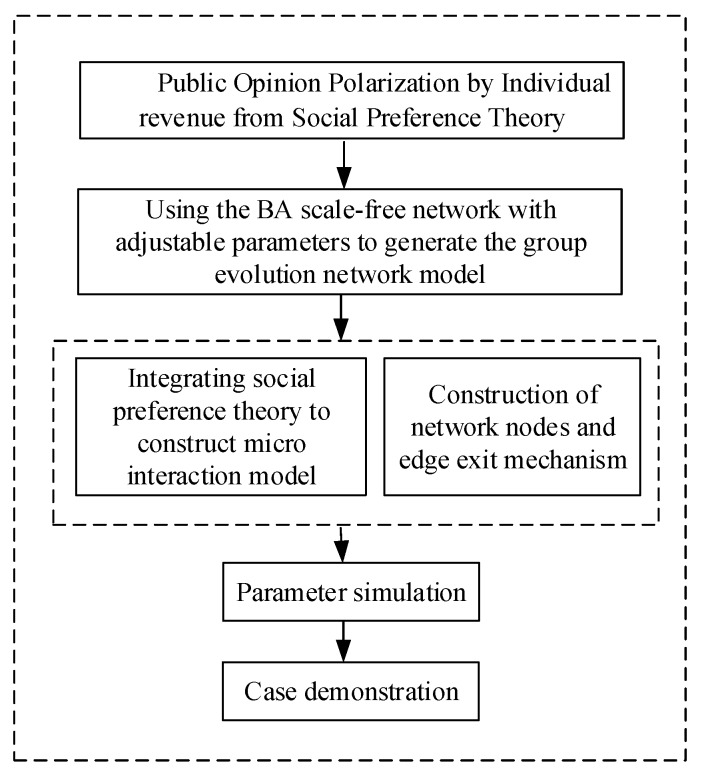
Research framework in this paper.

Based on the multi-agent method of Monte Carlo, we take individuals participating in interactions as agents [35]. Also, the network scale is set as *N*, meaning there are *N* Internet users (network nodes) in the network. Each individual’s attitude value in the network is represented by a number in the continuous interval [−1,1], and his/her initial attitude value obeys the random value of a uniform distribution. The specific research framework is shown in Figure 1:

For agent *i*, he/she will generate certain contentment if his/her view is affirmed when he/she interacts with other individuals in the network. The benefits brought by such contentment will prompt agent *i* to keep interacting with others. However, when agent *i* has a significantly different view with others, he/she will feel frustrated and create negative rewards due to being questioned. Based on this, the revenue generated by each interaction of agent *i* will vary with the views of its interlocutors. Assuming that at any moment, the total revenue generated by each interaction of agent *i* is *U*(*i*), which is composed of three parts, i.e., social mainstream revenue *U_s_*(*i*), viewpoint expression revenue *U_o_*(*i*), and individual interaction revenue *U_f_*(*i*). The corresponding equation can be expressed as follows.

*U*(*i*) = α*U_s_(*i*) + β*U_f_(*i*) + γ*U_o_(*i*) (1)

where *α*, *β*, and *γ* are influence parameters, which represent the influence degree for agent *i*’s social preference, neighboring preference, and self preference, respectively.

### 3.1. Social Mainstream Revenue U_s_

Although public opinion polarization usually evolves into the form of bi-polarization or single polarization, this paper mainly studies the bi-polarization phenomenon, that is, the emergence of two extreme views. When the bi-polarization phenomenon occurs, two opposite attitudes appear in the network, and they are totally against each other. Based on this, when measuring the social mainstream revenue of the network polarization phenomenon, it is not accurate to calculate the average attitude value of all individuals in the network. In this paper, the attitude values of the groups with positive and negative attitudes are firstly distinguished, and then the average attitude values of two different groups are calculated, respectively, namely, the average attitude value of individuals with negative attitude tendency *X_N_* and the average attitude value of individuals with positive attitude tendency *X_P_*.

If the individual attitude value *x_i_* in the network is uniformly distributed, then all *x_i_* ∈ [−1,1], individuals belonging to the interval [−1,0] are negative, while ones belonging to the interval (0,1] are positive. The calculation processes of the mean value of negative attitude $X_{N}$ and the mean value of positive attitude $X_{P}$ are as follows:

(2) $$X_{N}=\sum_{i=1}^{N}x_{i}\;x_{i}\in [-1,0]$$

(3) $$X_{P}=\sum_{i=1}^{N}x_{i}\;x_{i}\in (0,1]$$

**Figure 2:**
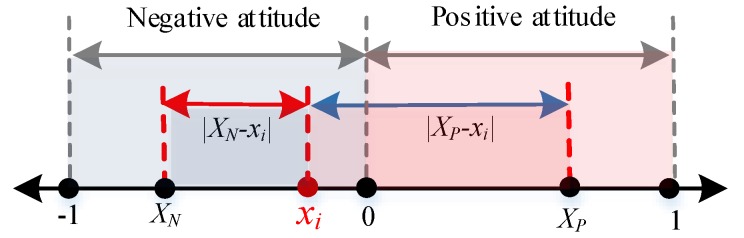
Diagram of social mainstream revenue with negative attitude.

Individuals often want to be recognized by other individuals through interaction behaviors due to their sociality. When the attitude value of agent *i* is *x_i_* ∈ [−1,0], agent *i* feels a negative attitude and he/she is more likely to be recognized by individuals with the same negative attitudes, while ones with positive attitudes will repel his/her view. The specific calculation is shown in Figure 2 and described by Equation (4).

(4) $$U_{s}(i)=\cos(\frac{\pi }{2}*|X_{N}-x_{i}|)\;+\;\varepsilon \cos(\frac{\pi }{2}*|X_{P}-x_{i}|)$$

where *ε* is an influence parameter, referring to the degree of individual aversion to deviation from the mainstream view of the society.

We can see from Figure 2, when individual *i* takes a negative attitude, the attitude distance ${|X}_{N}-x_{i}|$ between *i* and the mean negative attitude $X_{N}$ is within the interval [0,1], while the attitude distance ${|X}_{P}-x_{i}|$ between *i* and the mean positive attitude $X_{P}$ is within the interval [1,2]. At this point, the closer the attitude value of agent *i* and $X_{N}$ is, the more likely he/she will be affirmed by the individuals in the negative team. In other words, the smaller the value ${|X}_{N}{-x}_{i}|$ is, the larger the revenue is and all are positive, representing that the growth trend of agent *i* is similar to that of the cosine function within the interval [0,$\frac{\pi }{2}$]. Also, the greater the difference between the attitude value of agent *i* and $X_{P}$ is, the more likely he/she will be opposed by individuals in the positive attitude group. That is, the larger the value ${|X}_{P}-x_{i}|$ is, the smaller the revenue is and all are negative, representing that the growth trend is similar to that of the cosine function within [$\frac{\pi }{2}$, π].

**Figure 3:**
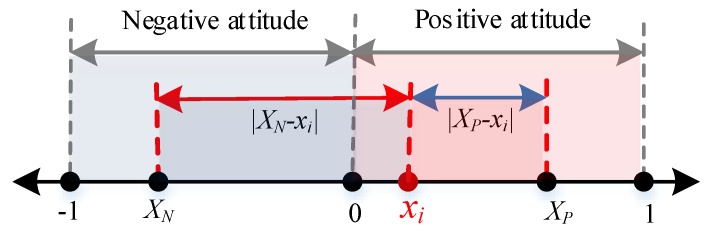
Calculation diagram of social mainstream revenue with positive attitude.

When *x_i_* ∈ [0,1], agent *i* has a positive attitude, which is more likely to be recognized by individuals with positive attitudes and repelled by ones with negative attitudes, as shown in Figure 3. The concrete calculation process is represented by Equation (5).

(5) $$U_{s}=\cos(\frac{\pi }{2}*|X_{P}-x_{i}|)+\varepsilon \cos(\frac{\pi }{2}*|X_{N}-x_{i}|)$$

Considering the only difference of the Equations (4) and (5) is the position of the parameter *ε*, we combine these two equations into one and add the conditions indicating which one will be used, as shown in Equation (6).

(6) $$U_{s}\left(i\right)=\left\{\begin{array}{c} \cos(\frac{\pi }{2}{*|X}_{N}-x_{i}|)+\varepsilon \cos(\frac{\pi }{2}{*|X}_{P}-x_{i}|),\;\mathrm{if}\;x_{i}\in \left[-1,0\right]\\ \cos(\frac{\pi }{2}{*|X}_{P}-x_{i}|)+\varepsilon \cos(\frac{\pi }{2}{*|X}_{N}-x_{i}|),\;\mathrm{if}\;x_{i}\in \left(0,1\right] \end{array}\right\}$$

### 3.2. Viewpoint Expression Revenue U_o_

When individuals come into contact with some hot information, they desire to express their own opinions, which will promote interaction among individuals. The revenue is acquired due to the successful expression of individual opinions after interactions, which is defined as the viewpoint expression revenue *U_o_* in this paper. For individuals, if their attitudes are closer to an extreme value, their expression willingness will be stronger, and the benefits will also be higher. In this paper, the range of attitude value is defined as a number in the interval [−1,1]. Since the function *y* = |*x*| is a line with the *Y*-axis as the symmetry axis and grows on both sides of the *X*-axis, and it is similar to the revenue function of viewpoint expression defined in this paper;here we use Equation (7) to calculate *U_o_*.

*U_o_*(*i*) = |*x_i_*| (7)

### 3.3. Individual Interaction Revenue U_f_

While agent *i* is interacting with agent *j*, *U_f_* will be produced. On the one side, individual *i* gains more when interacting with familiar individuals than when interacting with unknown ones. On the other hand, individuals will benefit more from interacting with those who share similar opinions than those who do not. Based on this, the benefits obtained from the interaction between two individuals are related to the intimacy degree *h_ij_* as well as friendliness degree *g_ij_*, and the corresponding equation is as follows.

*U_f_*(*i*) = h_ij_*g_ij_ (8)

The detailed calculation processes of *h_ij_*, as well as *g_ij_*, are illustrated below.

#### 3.3.1. The Calculation of the Intimacy Degree *h_ij_*

**Figure 4:**
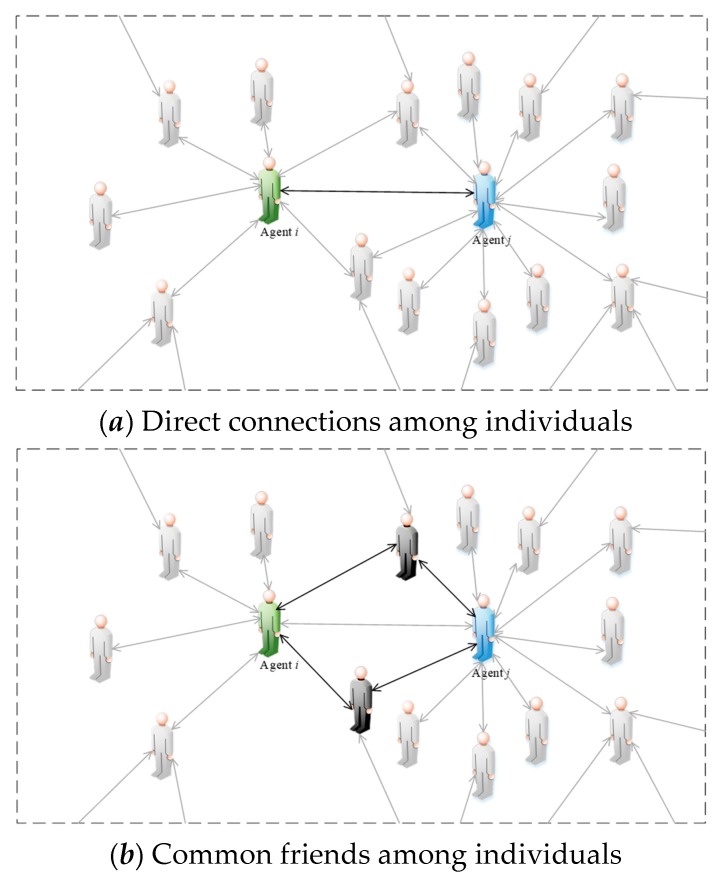

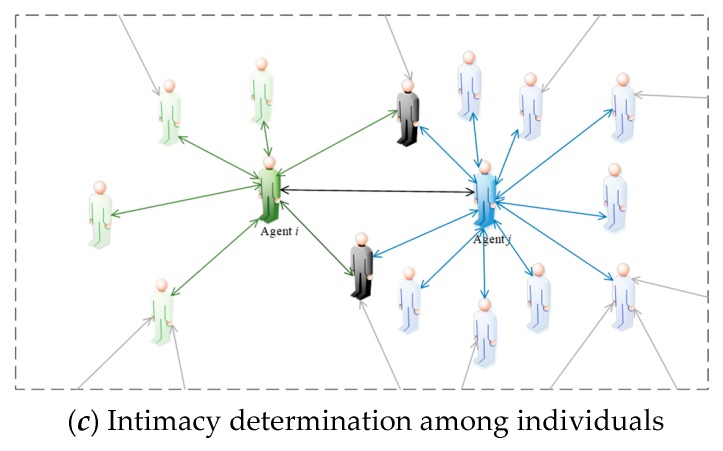
Diagram of intimacy *h_ij._*

The intimacy among individuals is used to represent the interconnections in a network. As shown in Figure 4, humanoid nodes represent interactive individuals in a network and edges denote the connections among individuals.

From Figure 4a, agent *i* and agent *j* are directly connected (see black lines in Figure 4a), meaning there is an interaction between them. Similarly, in Figure 4b, among all individuals connected with both agent *i* and agent *j*, there are two common connected individuals (see black humanoid nodes and black lines in Figure 4b), indicating that these two individuals have interactions with agent *i* and agent *j* at the same time; that is, these two individuals are the mutual friends of agent *i* and agent *j*. In a social network, the phenomenon that “friends of friends are your friends” often occurs, and two individuals with more common friends tend to be closer than two ones with fewer common friends. Therefore, we assume that the intimacy between two individuals is positively correlated with the number of mutual friends. However, as shown in Figure 4c, since the number of individuals connected with agent *j* (see blue lines in Figure 4c) is larger than that connected with agent *i* (green line), agent *i* and agent *j* have different intimacy degrees in spite of the same number of common friends between them. Assuming that the number of individuals connected to agent *i* in a network is *N_i_*, and also, the number connected to agent *j* is *N_ij_* in *N_i_*(*N_ij_* = *N_ji_*), then the intimacy degree of agent *i* and agent *j*, *h_ij_*, is expressed as follows:

*h_ij_* = *N_ij_*/*N_i_* (9)

Notice that due to *N_i_* ≠ *N*, we can find *h_ij_* ≠ *h_j_*. In addition, for the sake of simple calculation, we also assume that all connections among individuals in a network are bidirectional ones. Only two individuals with direct connection need to calculate intimacy. If there is no direct connection, the intimacy is 0.

#### 3.3.2. The Calculation of the Friendliness Degree *g_ij_*

Due to the difference of attitude values between agent *i* and agent *j*, the revenues obtained from the interactions are also different. Individuals usually show more friendliness to people with similar opinions. It is close to the descending trend of cosine function in [0,π]; that is, the larger the attitude difference |*x_i_* − *x_j_*| is, the lower the degree of friendliness is. When the attitude difference value falls into a certain threshold, the friendliness degree is positive; when it reaches a threshold, the friendliness degree is negative, and it continues to decrease with the increase of attitude difference. We assume that the attitude value is within the interval [−1,1], so the attitude difference |*x_i_ − x_j_*| is within the interval [0,2]. As a result, the multiplication coefficient $\frac{\pi }{2}$ is introduced to restrict the range of the friendliness. In doing so, *g_ij_* is defined as follows.

(10) $$g_{ij}=\;\cos(\frac{\pi }{2}*|x_{i}-x_{j}|)$$

### 3.4. The Total Interaction Revenue U

According to the above analysis, the total interaction revenue of agent i, U(i), can be expressed as follows:

(11) $$\begin{array}{c} U(i)\;=\;\alpha *U_{s}(i)\;+\;\beta *U_{f}(i)\;+\;\gamma *U_{o}(i)\\ =\{\begin{array}{l} \alpha (\cos(\frac{\pi }{2}{*|X}_{N}-x_{i}|)\;+\;\varepsilon \cos(\frac{\pi }{2}{*|X}_{P}-x_{i}|))+\beta \frac{N_{ij}}{N_{i}}*\cos(\frac{\pi }{2}*|x_{i}-x_{j}|)\;+\;\gamma |x_{i}|,\;x_{i}\in \left[-1,0\right]\\ \alpha (\cos(\frac{\pi }{2}{*|X}_{P}-x_{i}|)\;+\;\varepsilon \cos(\frac{\pi }{2}{*|X}_{N}-x_{i}|))\;+\;\beta \frac{N_{ij}}{N_{i}}*\cos(\frac{\pi }{2}*|x_{i}-x_{j}|)\;+\;\gamma |x_{i}|,\;x_{i}\in \left(0,1\right] \end{array}\} \end{array}$$

### 3.5. The Adoption of Social Preference Theory

Since each individual does not exist lonely in society, the revenues of individuals are related not only to themselves but also to others in many cases. When the total interaction revenue of individual *i* is a fixed value *U*(*i*), if he/she finds that the revenue of other individuals in the network is much lower or higher than *U*(*i*), recognition of individual *i* in the network are different in these two kinds of situations. Hence, there exists a weak correlation between the final revenue of individual *i*, *U*(*i*), and the revenue of interactive object, *U*(*j*). Nevertheless, individuals with different preferences have different recognition degrees. Social preference theory holds the view that individuals in the network can be roughly divided into three classes: individuals with an egoistic preference, who only care about their own income; individuals with an altruistic preference, who more concerned about other’s gains; and individuals with a fair preference, who only focus on allocation fairness. Based on the social preference theory, the calculations of the individual interaction revenue and the intimacy degree are modified in the following subsections.

#### 3.5.1. Modification of the Total Individual Revenue

Based on social preference theory proposed by Fehr and Schmidt [5], the revenue function of individuals with above three preferences is modified as follows:

(1) When individual *i* is with egoistic preference, he/she will care more about his/her own profit. Therefore, when the profit of individual *i* is less than that of individual *j* who interacts with him/her, he/she will reduce part of the revenue due to jealousy, and the influence degree related to their revenue difference |(*U*(*j*) − *U*(*i*))| is shown as follows:

(12) $$U_{P}(i)=U(i)-\varphi *\max\{(U(j)-U(i)),0\}$$

(2) When individual *i* is with altruistic preference, he/she will care more about an interlocutor’s benefit. Therefore, when the benefit of individual *i* is more than that of individual *j* who interacts with him/her, he/she will reduce part of the revenue due to sympathy. The equation is illustrated as

(13) $$U_{P}(i)=U(i)-\omega *\max\{(U(i)\;-\;U(j)),0\}$$

(3) When individual *i* is with fair preference, he/she will care more about allocation fairness. Therefore, when the benefit of individual *i* is more than that of individual *j* who interacts with him/her, he/she will reduce part of the revenue due to sympathy, and vice verse. The detailed calculation is shown in Equation (14)

(14) $$U_{P}(i)\;=\;U(i)\;-\;\varphi *\max\{(U(i)\;-\;U(j)),0\}\;-\;\omega *\max\{(U(j)\;-\;U(i)),0\}$$

where φ, ω are influence parameters, respectively, indicating the degrees of reduced revenue of individual *i* affected by individual *j*.

#### 3.5.2. Modification of the Intimacy Degree

**Figure 5:**
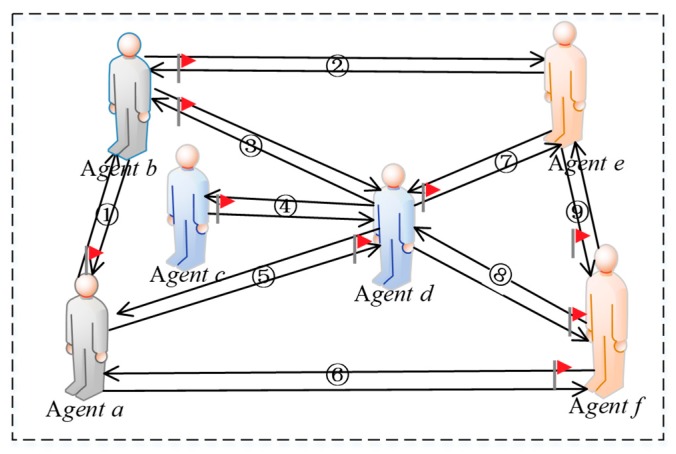
Comparison of interaction revenue.

In a network, netizens form connections through mutual attention, and thus build a virtual network. However, there are significant differences between online and offline social methods. First of all, the connections among individuals in a network are extremely unstable and the degree of intimacy between two individuals will be related to the benefits of their interaction. When an individual *i* interacts with *j* in a network, if the revenue he/she obtains is very small, then at the next moment, individual *i* will not want to interact with *j* anymore. Specifically, if the interaction intention of *i* to *j* is reduced, the intimacy of *i* to *j* will also be reduced. We believe that there exists a positive correlation between willingness to interact and intimacy. Secondly, due to the existence of heterogeneous preference individuals in a network, these individuals will present different manifestations when interacting shown in Figure 5.

In Figure 5, humanoid nodes represent interactive individuals in a network, where the gray nodes represent individuals with fair preference, the blue represent ones with egoistic preference, and the orange represent those with altruistic preference. In addition, serial numbers are used to represent different preferences, where “①” indicates interactions among individuals with fair preference, “④”describes interactions among individuals with egoistic preference, “⑨”represents interactions among individuals with altruistic preference, “②” and “⑥” represent interactions between individuals with fair preference and ones with altruistic preference, “③” and “⑤”denote interactions between individuals with fair preference and ones with egoistic preference, “⑦” and “⑧” indicate interactions between the individuals with egoistic preference and ones with altruistic preference. The red flag next to the humanoid node in Figure 5 indicates that the individual gains more by interacting than interlocutor. For example, in the interaction between agent *a* and agent *b*, there is a red flag on the side of agent *a*, denoting that the revenue of agent *a* is greater than that of agent *b*. Therefore, Figure 5 indicates that the quantitative relationships derived by comparing revenue with respect to individual interactions are as follows: *U*(*a*) > *U*(*b*), *U*(*b*) > *U*(*e*),*U*(*b*) > *U*(*d*), *U*(*c*) > *U*(*d*), *U*(*d*) > *U*(*a*), *U*(*f*) > *U*(*a*), *U*(*d*) > *U*(*e*), *U*(*f*) > *U*(*d*), *U*(*f*) > *U*(*e*).

**Figure 6:**
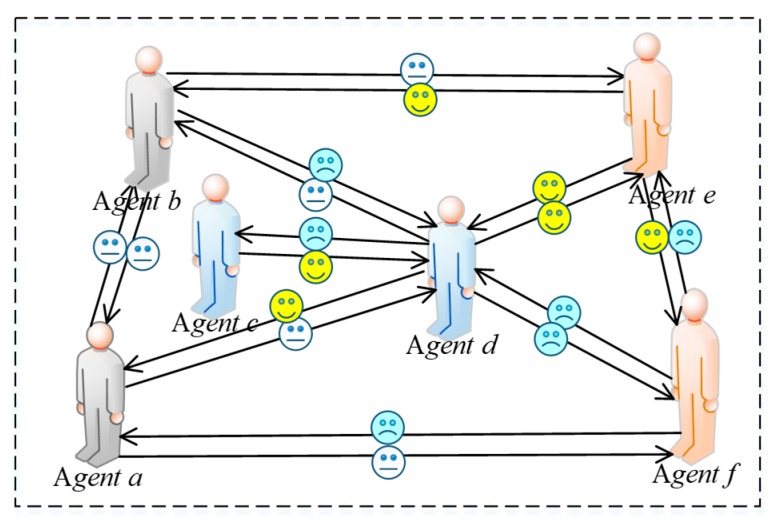
Revenue change diagram.

**Figure 7:**
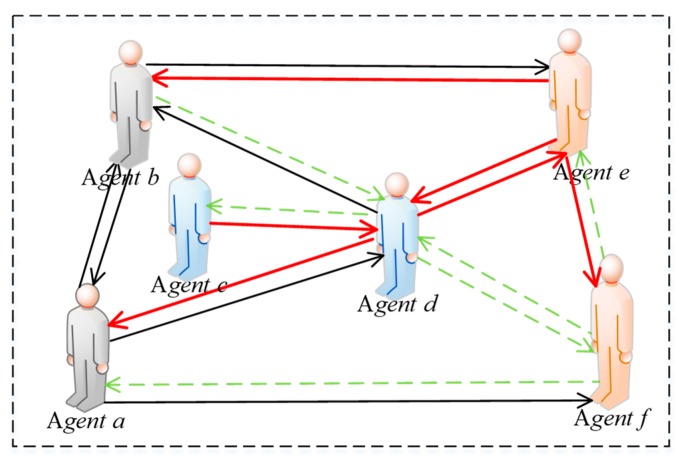
Intimacy modification diagram.

In addition, as shown in Figure 6 and Figure 7, agents *a* and *b* with fair preferences reduce their interaction intentions only when a big revenue gap is found between them, so their intimacy degrees are not easily changed. Whereas, when agent *c* interacts agent *d* with egoistic preference, since the benefit of agent *c* is greater than that of *d*, he/she will increase the willingness to interact with agent *d* again, and the intimacy degree *h_cd_* will be also improved. Conversely, agent *d* will reduce its willingness to interact with agent *c* again because his/her revenue is not as large as agent *c*, resulting in a decrement of intimacy degree *h_dc_*. Furthermore, the changes of intimacy degree between agent *e* and agent *f* with altruistic preferences are completely opposite to individuals with egoistic preferences. When the revenue of agent *e* with altruistic preference is less than that of interactive object *f*, the interaction intention of agent *e* with agent *f* will increase, rising intimacy degree *h_ef_*. However, agent *f* with altruistic preference reduces its intimacy degree with agent *e* because his/her own revenue is greater than agent *e*. After several interactions, if the intimacy degrees drop to 0 between the two interactive agents, the connection between them will be disconnected and no interaction will be conducted. In Figure 6, the icons of crying face, smiling face and expressionless face indicate the increment, decrease and invariability of interaction intention, respectively. In Figure 7, the solid red arrow, the dotted green arrow and the solid black arrow indicate the increment, decrease, and invariability of intimacy increases, respectively.

Through the above analysis, the intimacy function is modified as follows.

(1) Case 1, agent *i* with egoistic preference: the intimacy degree of agent *i* to agent *j* at time *t* + 1 is related to their previous intimacy degree and the revenue difference |(*U*(*j*) − *U*(*i*))| at time *t*. If the revenue of agent *i* at time *t* is less than agent *j*, the intimacy degree of agent *i* to agent *j* will be reduced due to jealousy, and vice versa, as shown below:

(15) $$h_{ij}\left(t+1\right)=\left\{\begin{array}{c} h_{ij}\left(t\right)-\frac{U\left(j\right)-U\left(i\right)}{U\left(i\right)}*h_{ij}\left(t\right),\;U\left(i\right)\neq 0\;\\ h_{ij}\left(t\right),\;U\left(i\right)=0 \end{array}\right\}$$

(2) Case 2, agent *i* with altruistic preference: the intimacy degree of agent *i* to agent *j* at time *t* + 1 is related to their previous intimacy degree and the revenue difference |(*U*(*j*) − *U*(*i*))| at time *t*. If the revenue of agent *i* at time *t* is more than agent *j*, the intimacy degree of agent *i* to agent *j* will be increased due to sympathy, and vice versa, as shown below:

(16) $$h_{ij}\left(t+1\right)=\left\{\begin{array}{c} h_{ij}\left(t\right)-\frac{U\left(i\right)-U\left(j\right)}{U\left(i\right)}*h_{ij}\left(t\right),\;U\left(i\right)\neq 0\;\\ h_{ij}\left(t\right),\;U\left(i\right)=0 \end{array}\right\}$$

(3) Case 3, agent *i* with fair preference: the intimacy degree of agent *i* to agent *j* at time *t* + 1 is related to allocation fairness. If revenue difference |(*U*(*j*) − *U*(*i*))| at time *t* is large, intimacy degree of agent *i* to agent *j* will be reduced, as shown below:

(17) $$h_{ij}\left(t+1\right)=\left\{\begin{array}{c} h_{ij}\left(t\right)-\frac{\max\left\{U\left(j\right)-U\left(i\right),0\right\}}{U\left(i\right)}*h_{ij}\left(t\right),\;U\left(i\right)\neq 0\;\mathrm{and}\;\left|U\left(j\right)-U\left(i\right)\right|>ɱ\\ h_{ij}\left(t\right),\;others \end{array}\right\}$$

where ɱ is a threshold, indicating the maximum acceptable revenue difference of agent *i* with an interlocutor.

In a sudden hot event diffusion process, individuals judge whether they can obtain certain revenue through interactive behaviors at the initial moment, so as to promote the continuation of interactive behaviors. With the changes in public opinion in the network, the interactive benefit of individuals will also change. When the individual revenue is negative, there will no longer be the driving force for interaction, and thus he/she will not focus on the hot event. Specifically, at the next moment, the individual will not interact with other individuals and become an isolated one in a network. The specific evolution steps of the whole interaction process are as follows:

Step 1: Build the initial network. The number of the initial network nodes is *m*_0_, and the nodes are connected randomly.

Step 2: Determine the growth mode of network nodes. *m* nodes are added to the network each time, connecting *m*_0_ nodes in the initial network. In other words, *m* edges are added each time, and the connection probability of the nodes in the initial network is positively correlated with the original node degree.

Step 3: Repeat the above operations until the number of nodes in the network increases to *N*, and the network diagram is undirected.

Step 4: Randomly select agent *i* and agent *j* to interact.

Step 5: Calculate *U_i_* and *U_j_* according to Equation (11).

Step 6: Compute revenue of agent *i* with different social preferences based on Equations (12)–(14).

Step 7: Renew intimacy function *h_ij_* of agent *i* to agent *j*.

Step 8: Judge whether the cumulative revenue of agent *i* in the network is greater than 0. If the revenue is greater than 0, the interaction will continue. If not, all connections of agent *i* in the network will be disconnected, making him/her be an isolated node.

Step 9: Repeat Steps 4–8 until the end of evolution.

**Figure 8:**
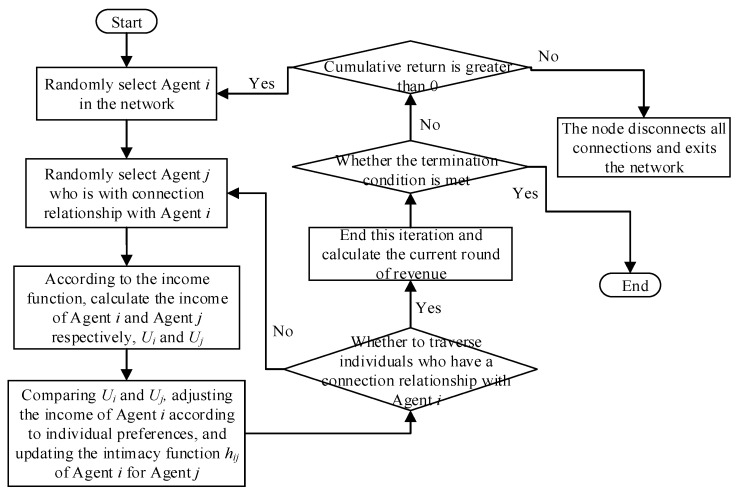
Simulation process of opinion evolution.

The specific simulation process of this paper is shown in Figure 8:

## 4. Simulation Experiment

This section analyzes the influences of different social preferences and individual benefits on public opinion polarization effects.

### 4.1. The Influences of Different Social Preferences on Public Opinion Polarization Effects

**Figure 9:**
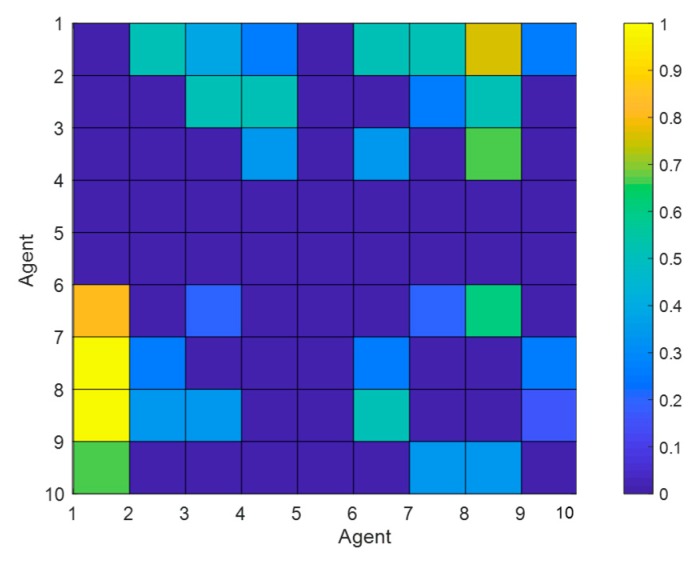
The mottled diagram of the intimacy degree of all nodes.

**Figure 10:**
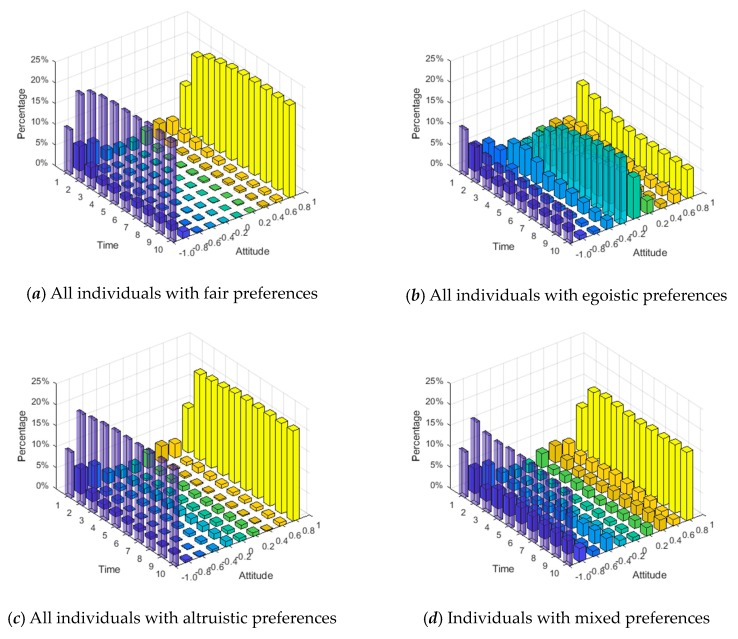
Attitude values of individuals at different times.

**Figure 11:**
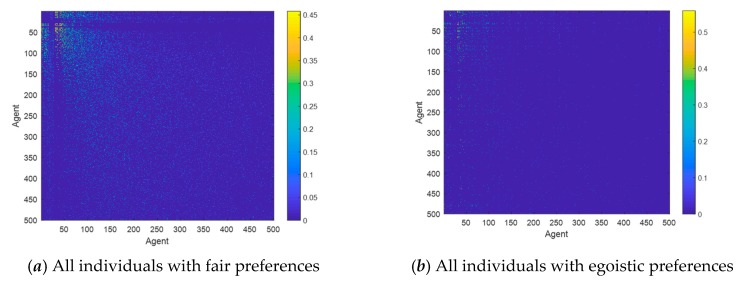

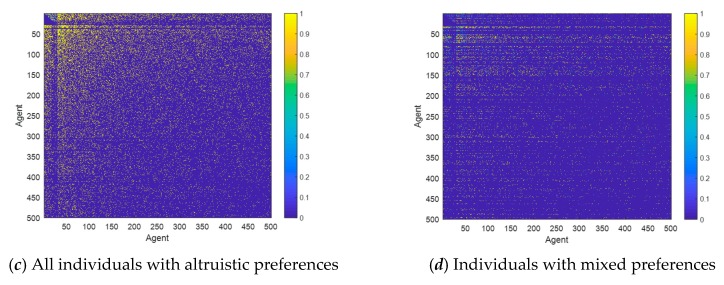
Distributions of intimacy degree.

This section conducts numerical simulation experiments to study the influences of individuals with three different preferences on the public opinion polarization effects. The network is constructed by extended BA scale-free network [32], and other simulation parameters are as follows: *N* = 500, φ = 0.5, ω = 0.5, *α* = 0.32, *β* = 0.6, *γ* = 0.05. The specific network characteristic structure is shown in Table 1. Figure 9 is a mottled diagram of the intimacy of all nodes in the network at the initial moment according to Equation (9), and the specific calculation results are shown in Equation (18). Figure 10 and Figure 11 respectively represent attitudes of individuals and distributions of intimacy degree after the occurrence of opinion polarization phenomenon, when considering four different situations, i.e., all individuals with the same fair preferences, individuals with the same egoistic preferences, individuals with the same altruistic preferences, and individuals with mixed preferences where each preference accounts for 1/3. In Figure 10, a three-dimensional bar chart is shown, where the *x*- and *y*-axes represent the distributions of interaction time and attitude values, respectively, and the *z*-axis represents the proportion of individuals in different attitude intervals at different times. In Figure 11, the concrete intimacy degree values are also shown in the right color bar of this figure. In this simulation, when time is 6, the public opinion polarization phenomenon appears. This is because attitudes of all individuals in the network do not change at time = 6, and the vast majority of individual attitudes are in extreme values (i.e., |*x_i_*| > 0.9).

(18) $$h=[\begin{array}{cccccccccc} 0 & 0.5000 & 0.3750 & 0.2500 & 0 & 0.5000 & 0.5000 & 0.7500 & 0.2500 & 0.2500\\ 0 & 0 & 0.5000 & 0.5000 & 0 & 0 & 0.2500 & 0.5000 & 0 & 0\\ 0 & 0 & 0 & 0.3333 & 0 & 0.3333 & 0 & 0.6667 & 0 & 0\\ 0 & 0 & 0 & 0 & 0 & 0 & 0 & 0 & 0 & 0\\ 0 & 0 & 0 & 0 & 0 & 0 & 0 & 0 & 0 & 0\\ 0.8000 & 0 & 0.2000 & 0 & 0 & 0 & 0.2000 & 0.6000 & 0 & 0.4000\\ 1.0000 & 2.5000 & 0 & 0 & 0 & 0.2500 & 0 & 0 & 0.2500 & 0\\ 1.0000 & 0.3333 & 0.3333 & 0 & 0 & 0.5000 & 0 & 0 & 0.1667 & 0.3333\\ 0.6667 & 0 & 0 & 0 & 0 & 0 & 0.3333 & 0.3333 & 0 & 0\\ 0.6667 & 0 & 0 & 0 & 0 & 0.6667 & 0 & 0.6667 & 0 & 0 \end{array}]$$

From Figure 9 and Equation (18), in the network with the number of nodes *N* setting as 10, there are at most (10−1)^2^ = 81 edges except for himself/herself, and each edge corresponds to an intimacy value. For example, in Equation (18), the intimacy value at the matrix element *h*(1,2) is 0.5, indicating that the intimacy degree between agent 1 and agent 2, *h*_12_, is 0.5. In Figure 9, the color in the grid of the first row and the second column represents 0.5. If the value at element (*i*, *j*) in the matrix is 0, it means that agent *i* and agent *j* are not connected. At this point, the color of the grid in Figure 9 appears dark blue. Notice that all *h*(*i*,*i*) in the matrix’s diagonal line are meaningless and thus are represented by 0.

**Figure 12:**
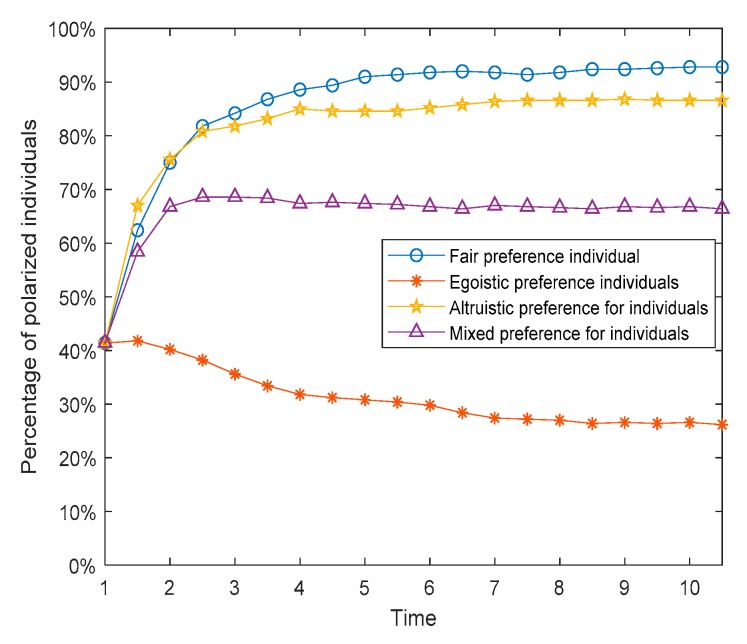
Percentage of polarized individuals over time.

In addition, from the intimacy distributions in Figure 11, when time = 6, there are great differences in the intimacy degrees among these three preference networks, wherein the altruistic preference network, the intimacy degrees are greater than those in the fair preference network, and the intimacy degrees in the fair preference network are greater than those in the egoistic preference network. Based on this, individuals with altruistic preferences have a greater willingness to interact in the network, and this willingness will be further increased after multiple interactions, which manifests the improvement of intimacy degrees. In contrast, individuals with egoistic preferences tend to reduce their willingness to interact after multiple interactions, which shows the reduction of intimacy degrees. Moreover, individuals with fair preferences fall somewhere in between. However, it can be seen from Figure 10 and Figure 12 that the polarization percentage (i.e., the proportion of the number of individuals of |*x_i_*| > 0.9 to all individuals) in the network composed of individuals with fair preference is the highest, followed by altruistic preference, mixed preference, and egoistic preference in descending order.

**Figure 13:**
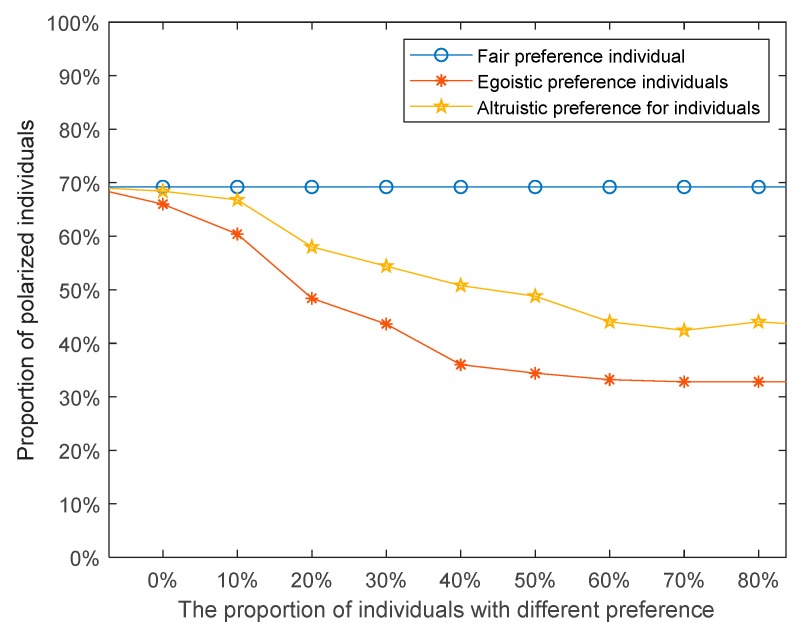
The relationships between percentage of polarized individuals and the proportion of individuals with different preferences.

Besides, in order to further study the influences of different social preferences on the effects of public opinion polarization, the proportions of individuals with three different preferences were set to 10%, 20%, 30%, 40%, 50%, 60%, 70%, 80%, 90%, 100% (the rest of the individuals are assumed to be without any preference). Analysis and discussion are focused on the setting of other parameters unchanged. The results are shown in Figure 13.

As shown in Figure 13, as the proportion of individuals with egoistic preference and altruistic preference in the network increases, the polarization percentage in the network decreases. In contrast, with the increase of the proportion of individuals with fair preference, the polarization percentage hardly changed.

**Figure 14:**
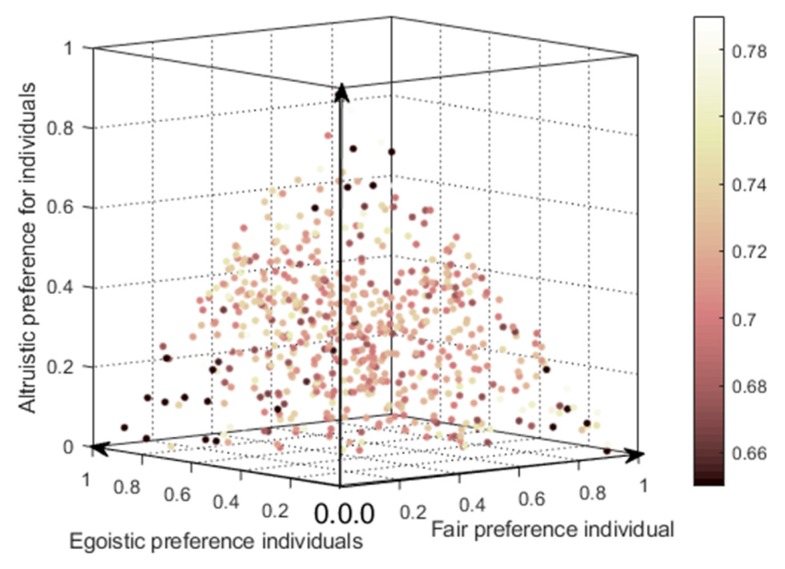
The influences of different preference proportions on the polarization after 100 experiments.

In order to understand the influences of different preference proportions on the polarization, the BA network is simulated for 100 times, and each test randomly generates different individual preference ratio and combines into different social networks (for example, setting egoistic preference accounting for 31%, altruistic preferences accounting for 33%, fair preference for 36% is one of the experiments) to calculate the polarization shown in Figure 14. Each scatter plot in this figure represents one simulation result, where the *x*-, *y*-, and *z*-axes denote the proportion of fair, egoistic, and altruistic preferences, respectively. The color of the scatter represents the polarizability, where the lighter the color is, the higher the polarizability, and vice verse. The results are shown in the color bar on the right in Figure 14.

Also, from Figure 14, since the sum of the proportions of three different preferences in the network equals to 1, the scattered points in this figure are all distributed in the same triangular section. The three vertices of the triangle represent the network polarization effects when only one social preference in the network exists. These three vertices show dark color, representing low polarizability, while the light color in the middle points indicates high polarizability. Simulation results show that opinion polarization is low when there is only one social preference in the network. In addition, the change trend of polarizability shown in Figure 14 is slightly different from that in Figure 13. This is because, in Figure 13, when analyzing the influences of the proportion of individuals with different preferences on the public opinion polarization effects, other individuals are treated as ones without preferences, that is, revenue is not adjusted according to their preferences. In the three-dimensional diagram shown in Figure 14, if the proportion of individuals with one preference increases, the proportion of individuals with other preferences decreases accordingly so that the sum of the three equals to 1.

**Figure 15:**
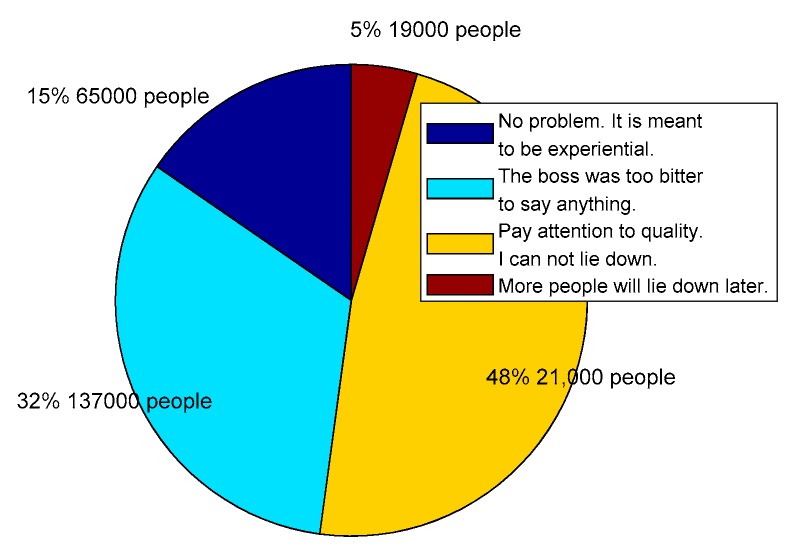
Opinion distributions of “Sleep”, “Cool” events.

For example, in real life, the main distributor of household goods “IKEA Group Corporate” stores often witnesses people who sit or lie down in the store exhibition area, which has been criticized by netizens. On 22 August 2019, these uncivilized behaviors caused heated discussions on the Sina blog. According to the statistics, until 25 August 2019, a total of 422,000 netizens participated in the event to vote, and the results were shown in Figure 15.

Figure 15 demonstrates that although most netizens who participated in the voting thought that they should not have too much rest in IKEA, they preferred to view problems from their own perspectives. However, 15% of netizens still thought this kind of behavior was not a problem, and they preferred to view the problem from the perspective of customers, while 32% of netizens were concerned about whether IKEA’s owner would lose money, preferring to view from the seller’s perspective. Others were worried that more people would lie down later. Thus, we can see that there are heterogeneous individuals in the network whose preferences are different. However, these individuals with different preferences widely exist in the network, and they may have different views on the same thing, which has a significant influence on the network polarization phenomenon.

### 4.2. The Analysis of Interaction Modes among Individuals with Different Preferences

The different preferences of individuals can affect the interaction revenue between individuals in the network, thus indirectly influencing the intention of the next interaction between them. Firstly, when the benefit of the interaction between individual *i* and *j* is always negative, then after several interactions, individual *i* will no longer be willing to interact with *j*. At this moment, the connection between individuals *i* and *j* is broken in the social network. Secondly, except for the individual *j*, individual *i* will also interact with other individuals in the network and obtain benefits from them. However, if the total gain obtained by individual *i* in interacting with these individuals is negative, then at the next moment, individual *i* will no longer be willing to participate in the interaction and express his/her own views. In the network, individual *i* is shown as *i* being completely disconnected from all nodes and becoming an isolated one, thus exiting the propagation process of the hot event. As a result, the different preferences of individuals can act on the network structure by influencing the benefits of individuals. Also, the network structure will also have an important impact on public opinion polarization, and the network with high aggregation will help increase the interaction frequency of individuals, thus affecting public opinion polarization.

**Figure 16:**
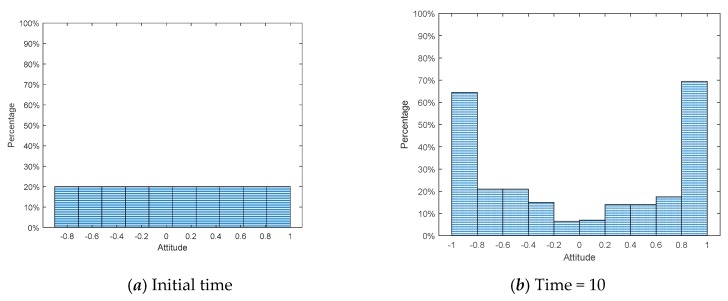
Distribution of attitude values at the initial time and time = 10.

**Figure 17:**
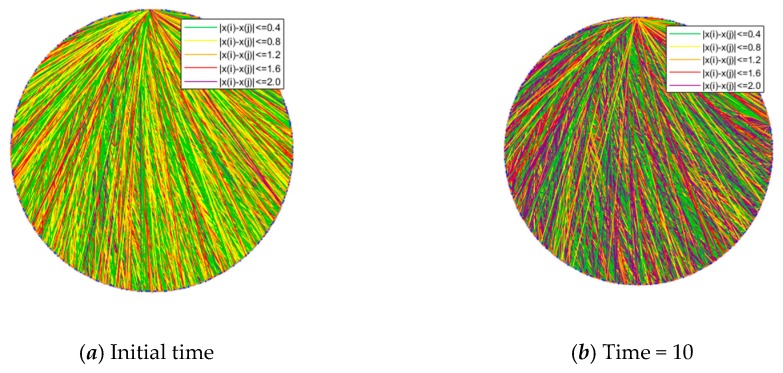
Connections among network nodes at the initial time and time = 10.

**Figure 18:**
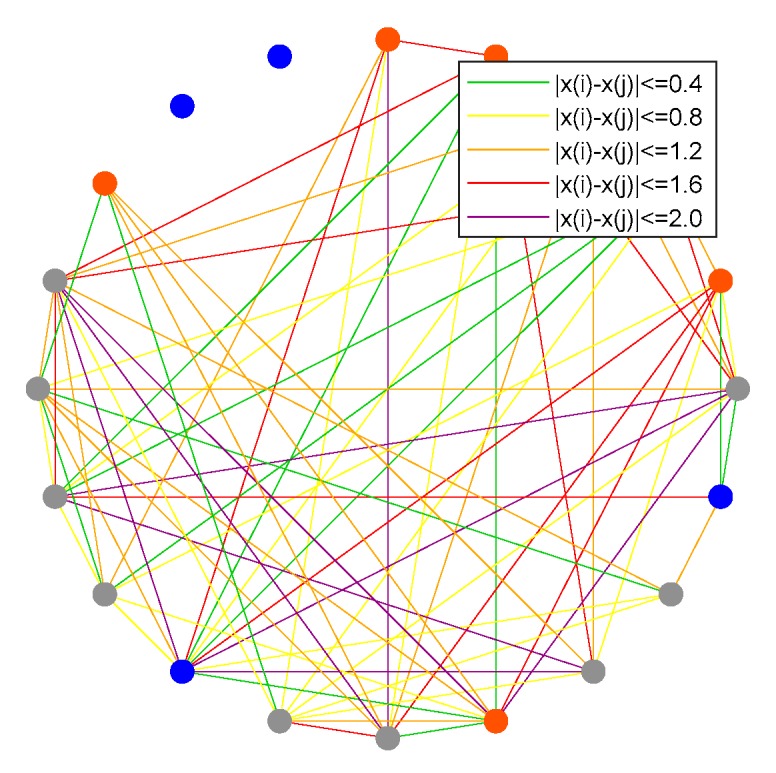
Node connections when *N* = 20.

**Figure 19:**
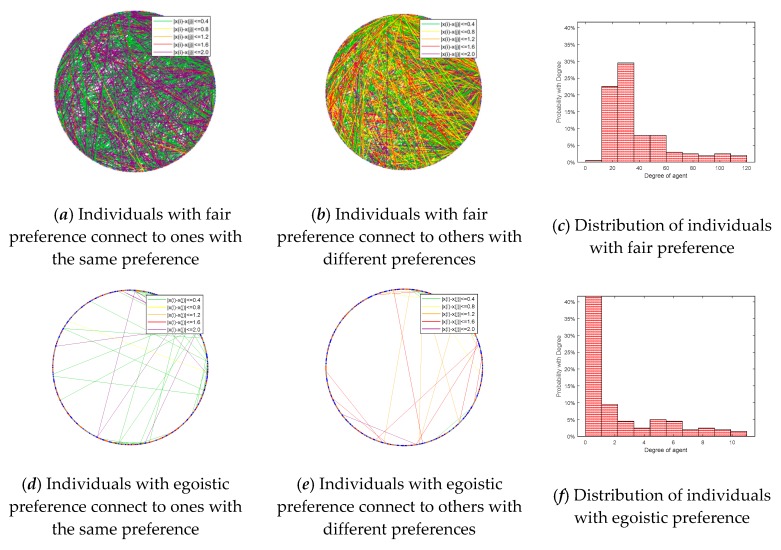

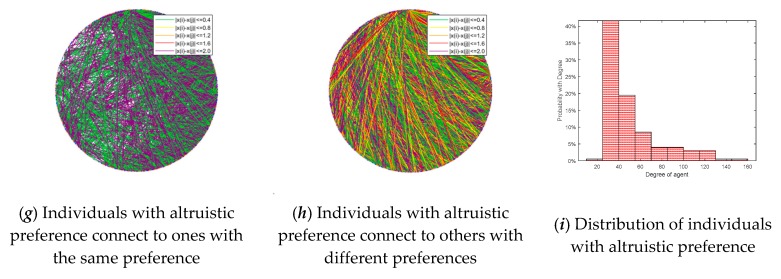
Connections of three different preferences and distribution.

This section compares connections at the initial moment and the moment after the emergence of public opinion polarization (time = 10). The results are shown in Figure 16, Figure 17, Figure 18 and Figure 19, where Figure 16 is the histogram of attitude distribution frequency in the network at the initial moment as well as time = 10, respectively, and Figure 17 shows the connections of network nodes at the two different moments. In order to better illustrate the specific meaning of Figure 17, a diagram of connections among nodes (*N* = 20) is drawn and shown in Figure 18. The network used in the experiment is the BA network; the network aggregation is 0.320, *N* = 500, and the remaining parameters are φ = 0.1, ω = 0.1, *α* = 0.35, *β* = 0.35, *γ* = 0.2. Each of the three preferences contributes 1/3 and they are mixed in the network.

From Figure 16, the attitude values of all individuals *x_i_* ~ *U*(−1,1) in the network at the initial moment are evenly distributed, while at time = 10, there is an obvious polarization, and the attitude values of most individuals are at the extremes of +1 and −1. Figure 17 shows the connections of network nodes at the initial time and time = 10. As shown in Figure 17 and Figure 18, three kinds of scatter points represent individuals with different preferences in the network: the grays catter points represent individuals with fair preference, the blue ones represent individuals with egoistic preference, and the orange ones represent individuals with altruistic preference. In these two figures, the lines represent the connection relationships among the scattered points. If there is a line between two scattered points, they are neighbors and can interact with each other, where the lines include five colors, i.e., green, yellow, orange, red, and purple. Since the attitude values are evenly distributed in the interval [−1,1] at the initial moment, the attitude distance between two individuals is relatively small, and |*x_i_* − *x_j_*| > 1.6 is less likely to occur. After the polarization phenomenon occurs, as shown in Figure 17b, the attitude values of all individuals in the network change significantly, and most individuals show green and purple lines, representing the two extremes of the attitude distances between adjacent individuals |*x_i_* − *x_j_*| < 0.4 or |*x_i_* − *x_j_*| > 2.0.

In addition, Figure 19 shows the connections and distribution of three different preferences in the network after the occurrence of public opinion polarization phenomenon, in which the lines and scatter points are represented in the same way as shown in Figure 17, that is, the scatter points of three different colors represent the individuals with different preferences, and the lines of different colors represent the different attitude distances among neighboring individuals. From the vertical comparisons in Figure 19, we can see that the connections among individuals with fair preference and altruistic preference change little in the network, while the connections among individuals with egoistic preference almost completely break. The horizontal comparisons in Figure 19 show that individuals with different preferences are more likely to interact. Figure 19a,d,g represents the interactions among individuals with the same preference, respectively. At this point, the attitude distance among individuals with the same preference almost only has two extreme values, |*x_i_* − *x_j_*| < 0.4 or |*x_i_* − *x_j_*| > 2.0. In contrast, the interactions among individuals with different preferences represented in Figure 19b,e,h appear more connections of intermediate attitude distances, which illustrates that after the emergence of public opinion polarization, the existence of more differentiated individuals is easier to stabilize the network. Moreover, Figure 19c,f,i shows the degree distribution of individuals with different preferences, in which the network degree distribution of egoistic individuals is significantly smaller than that of the other two preferences, while the difference between fair preference and altruistic preference is not obvious.

### 4.3. The Influence of Individual Revenue on Public Opinion Polarization

In Section 3, the revenue of individuals is divided into three parts: social mainstream revenue *U_s_*, viewpoint expression revenue *U_o_*, and individual interaction revenue *U_f_*. At the initial moment, after perceiving the information, individual viewpoint expression revenue *U_o_* will lead to generating a willingness to communicate, which drives the individual to keep interaction at the next moment. However, depending on the attitude distance among the interlocutors, *U_f_* will encourage each other due to the similarity of views, or argue with each other due to the repulsion of views, thus producing both positive and negative benefits to the interlocutors. Similarly, when calculating the mainstream income, *U_s_*, individuals will also generate negative revenue because they deviate from the mainstream view of the society. Therefore, if the revenue of each interaction is negative, the individual will tend to adjust his/her attitude to change the revenue he/she obtained, or he/she may lose interest in the event due to the negative revenue after adjusting his/her attitude, thus completely leaving the network. Hence, to analyze the public opinion polarization phenomenon, it is necessary to consider the change of individual profit in the network and the number of exit network nodes.

#### 4.3.1. The Influences of Different Preferences on Individual Revenue

**Figure 20:**
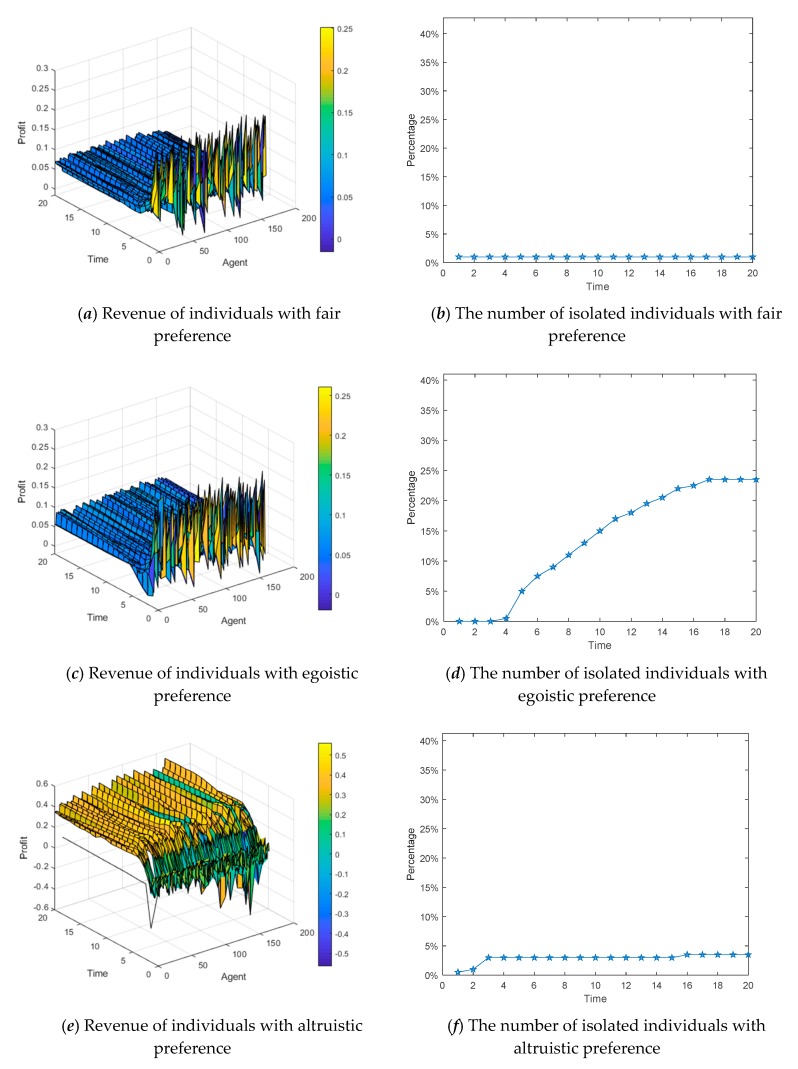
The influences of different preferences on individual revenue.

This section uses a BA network consisting of 600 nodes, in which each of the three different preferences accounts for 1/3 (i.e., each is 200) and is evenly distributed. The revenue differences between the three preferences in the network are observed in the experiment. Figure 20a,c,e shows the three-dimensional graph of individual revenue with changes over time, in which the *x*-axis represents the interaction time, the *y*-axis represents the number of the interaction individuals in the network, and the *z*-axis represents the revenue growths of individuals through interactive behaviors at different times. Figure 20b,d,f describes that the number of individuals with three different preferences that exit the viewpoint interaction and become isolated ones due to negative revenue changes over time.

We can see from Figure 20, individuals with altruistic preference gain the most through interactions. Individuals with fair preference becoming isolated ones are the least. In addition, the revenue of individuals with egoistic preference fluctuates greatly at the initial moment, and many individuals have negative revenue, so the probability of becoming isolated nodes is the highest. Through experiments, it is easy to find that individual revenue is continuously growing in the network. Most of the individuals in the network gain the benefits and also increase their revenue by adjusting their interactive strategies, which improves interaction willingness and leads to the emergence of several mainstream opinion groups.

#### 4.3.2. The Influences of Individual Revenue on Public Opinion Polarization

**Figure 21:**
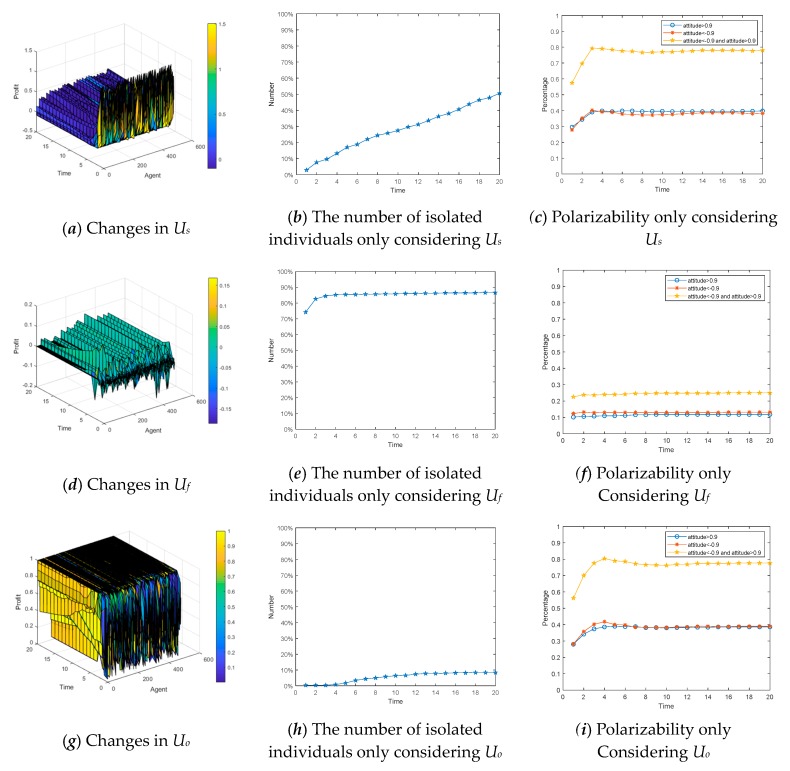
The influences of individual revenue on public opinion polarization.

Since the total individual revenue is composed of *U_o_*, *U_s_*, and *U_f_*, in order to analyze the influences of revenue on the polarization effect, we assume that there only exists one part of revenue in the network and observe the evolution of public opinion. Figure 21a,d,g represents changes in individual revenue only considering *U_o_*, *U_s_*, or *U_f_* in the network over time, respectively. Figure 21b,e,h describes the growths of isolated individuals in the network over time. Figure 21c,f,i denotes the growths of the polarizability in the network over time.

From Figure 21a–c, when individuals only gain benefits through social recognition, the benefits are relatively large at the initial time. However, with the further evolution of public opinion, polarization gradually emerges, and the growth trend of revenue begins to slow down. Moreover, when the revenue decreases, the number of isolated individuals exiting interactive behaviors starts to increase. In Figure 21d–f, when individuals can only benefit from the interaction, due to dispersion of views at the initial time, it is very difficult for them to find like-minded individuals to interact, so the profit is very low, making individuals unwilling to interact once again. Thus, the number of isolated individuals grows fast at the initial time.However, when time = 4, almost 85% of individuals are out of the communication of events, and at the same time, due to the weak interaction willingness, it is hard for the polarization phenomenonto appear. As can be seen from Figure 21g–i, when individuals can only get benefits by expressing their own opinions, they have strong desires to interact with each other, so the number of isolated individuals grows slowly. Most individuals are willing to express their own opinions in the network, so polarization is more likely to occur.

### 4.4. The Influences of Dynamic Changes of Individual Preferences on Public Opinion Polarization

**Figure 22:**
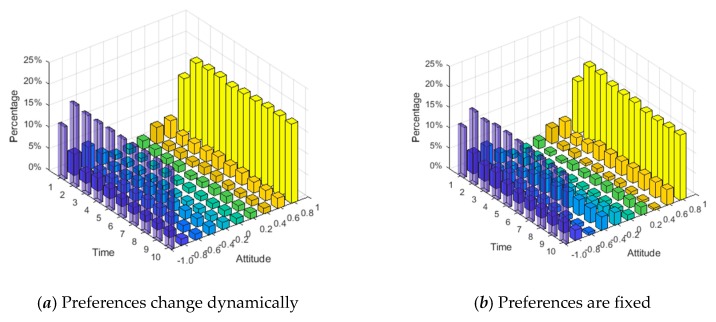
Individual attitude distributions at different times.

**Figure 23:**
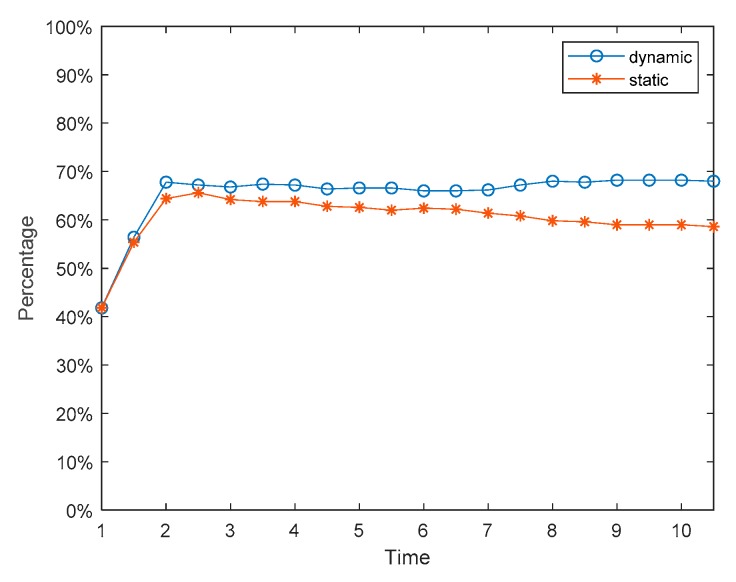
Polarization proportion with changes over time.

**Figure 24:**
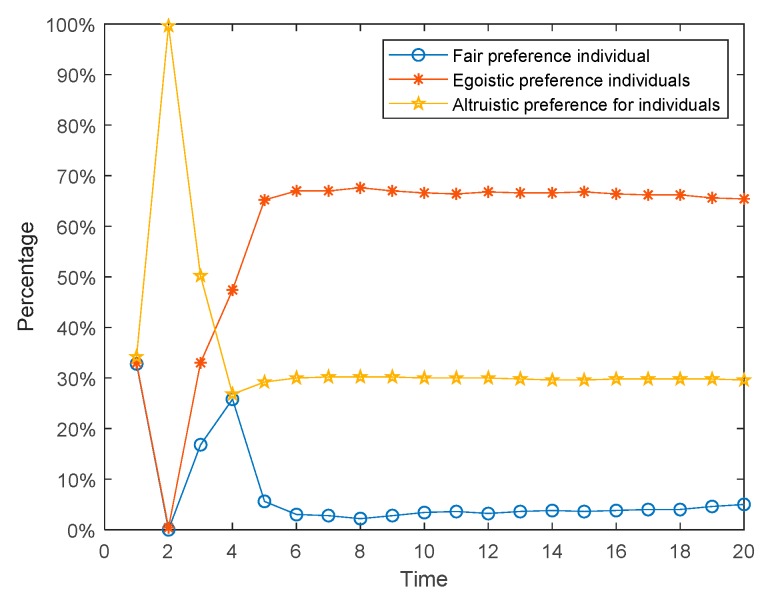
Individual proportions with three different preferences changing over time.

The social preferences of individuals discussed in Section 4.1, Section 4.2 and Section 4.3 are fixed, but in reality, they will change continuously with the interaction. Individuals with an egoistic preference at the beginning may hold the fair or altruistic preference in the later stage, while those who hold an altruistic preference may also change to the egoistic or fair preference later. Based on this, we assume that social preferences have dynamic variability and define rules as follows. When the revenue of the individual in the network is greater than the threshold *d*_1_, his/her preference will turn into altruistic preference. When the revenue of the individual in the network is less than the threshold *d*_2_, his/her preference will turn into egoistic preference. Similarly, when the revenue of the individual in the network is between the middle, his/her preference will convert into fair preference. The results are shown in Figure 22, Figure 23 and Figure 24. Figure 22 is three-dimensional histogram of individual attitude distributions. Figure 23 is line chart of polarization proportion with changes over time. Figure 24 is line chart of individual proportion with three different preferences changing over time.

It can be seen from Figure 22 and Figure 23 that, compared with the situation of fixed preferences, when the social preferences change dynamically, the individual polarization proportion will be larger. However, in Figure 24, at the initial moment of the public opinion evolution, almost all individuals with different preferences are converted into ones with fair preference, but this period is very short, and then individuals with fair preference gradually are changed into holding other preferences. When time = 6, the preferences of individuals in the network will no longer change and reach stability. At this point, compared with the initial moment, the number of individuals with egoistic preference increases sharply, the number of individuals with fair preference decreases significantly, and the number of individuals with altruistic preference does not decrease significantly.

## 5. A Real Case Study

In this section, the case of “Internet red beating pregnant women” is taken as an example to verify the model established in this paper. On 9 September 2018, a blogger with the microblog number of “Baytt” released a microblog as the victim, claiming that she had been pregnant for 32 weeks. When she went out, she met another Internet red named “Saya” walking with her dog. Because Saya’s dog rushed at her, she was unilaterally beaten due to their quarrel. The microblog spread in the network after being forwarded by official media such as “Headline News” and “pear video”, and quickly reached network opinion polarization. Almost everyone began to blame “Saya”. But soon after, as “Saya” apologized and explained her actions, more truth emerged. Netizens found that the truth of the incident was that the pregnant woman and her husband beat “Saya” first after the quarrel, while “Saya” did not beat the pregnant woman. At this time, public opinion began to reverse. Many netizens turned to support “Saya” and accused the pregnant woman, but some netizens still supported the pregnant woman.

**Figure 25:**
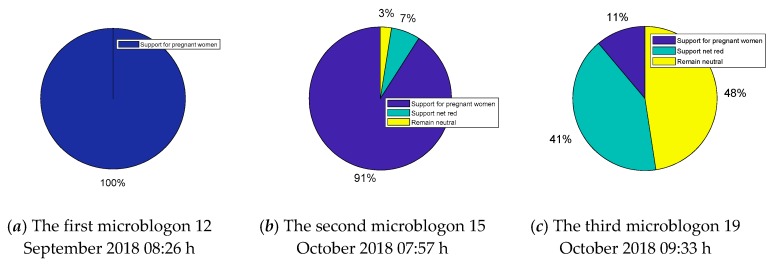
Opinion distributions of the event “web celebrity beating a pregnant woman”.

The incident of “Internet red beating pregnant women” attracted the attention of microblog bloggers “Headline News”, which has 68 million fans. “Headline news” released three microblogs about the incident on 12 September 2018 08:26 h, (the data collected can be found in the following link: https://weibo.com/breakingnews?is_all=1&is_search=1&key_word=%E7%BD%91%E7%BA%A2%E6%AE%B4%E6%89%93%E5%AD%95%E5%A6%87#1579968622052), 15 October 2018 07:57 h (the data collected can be found in the following link: https://weibo.com/breakingnews?is_all=1&is_search=1&key_word=%E7%BD%91%E7%BA%A2%E6%AE%B4%E6%89%93%E5%AD%95%E5%A6%87#1579968662315), and 19 October 2018 09:33 h (the data collected can be found in the following link: https://weibo.com/breakingnews?is_all=1&is_search=1&key_word=%E7%BD%91%E7%BA%A2%E6%AE%B4%E6%89%93%E5%AD%95%E5%A6%87#1579968683067), respectively. These microblogs are widely focused, with more than 10,000 comments per microblog.Therefore, in order to accurately restore the public opinion development process of the “Internet red beating pregnant women” incident, we separated the comments under these three microblogs of “headline news” and analyze them. Through the emotional orientation [36] in the comments, we roughly divided the comments we have separated into three categories: those who support pregnant women, those who support the internet red, and those who maintain neutrality, as shown in Figure 25:

As can be seen from Figure 25, in the first microblog, almost all netizens unilaterally supported the pregnant woman and criticized Internet red Saya’s behavior. However, in the second microblog released on 15 October, some neutral individuals appeared, and even a small number of netizens turned to support “Saya”. In the post published on 19 October, a large number of individuals supported “Saya” or remained neutral, while the proportion of individuals still supporting the pregnant women dropped to 11%. It can be seen that the hot event showed a reversal of public opinion. 

**Figure 26:**
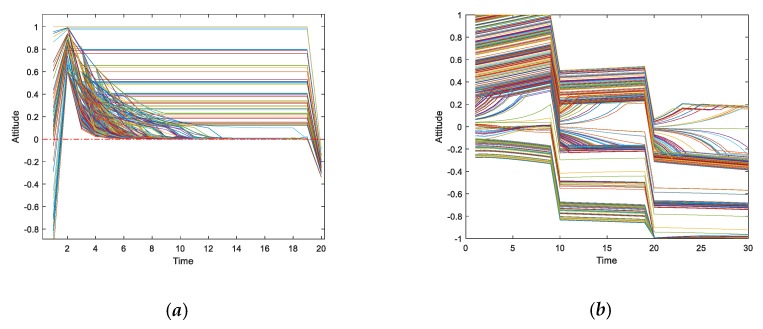
The results of a real case simulation of two models. (**a**) Model which considers individual heterogeneity preference in this paper. (**b**) Macro polarization model in [14].

However, it is worth noting that Internet users have obvious preferences in the public opinion communication of the network hot event. Because many netizens prefer to support the weak, when the information is not clear at the beginning, almost all of them supported pregnant women one way. However, with the truth gradually revealed, there were many individuals who still maintained their preferences and did not change their views. Therefore, we simulate the event using the public opinion polarization model which considers individual heterogeneity preference and compares the macro polarization model in [14]. The parameters we used in the simulation are as follows: *α* = 0.15, *β* = 0.8, *γ* = 0.05, the network is a BA scale-free network with clustering C = 0.324. The results are shown in Figure 26.

From Figure 26a, we can see that at the initial time, almost all individual attitudes are positive, that is, they support the pregnant woman’s viewpoint, but with the evolution of public opinion, some individuals’ attitudes begin to shift to neutrality. Finally, when time = 18, their attitudes change again, and most of the individual attitudes turn negative, that is, they support “Saya”, which is roughly consistent with the trend shown in Figure 25. At the same time, as shown in Figure 26b, we use the macro model proposed in [14] to simulate and get simulation results. Comparing these two figures, we can see that the evolutionary trends of these two are basically the same. Whereas, the model considering individual heterogeneity preference can polarize public opinion more quickly than the one proposed in [14], the reason may be that, in such a typical case as “Internet red beating pregnant women”, individuals have obvious preferences which make individuals with the same preference form similar viewpoints. For example, at the beginning of this incident, a very large percentage of people prefer to support the weak. After that, with the disclosure of information, most individuals hold fairness preference and supported Internet red. As the result, in the evolution of public opinion, the opinion polarization phenomenon can occur quickly, which is roughly consistent with the model proposed in this paper. However, the model proposed in [14] considers the dynamic conformity of individuals, which make individuals have a relatively low tendency to conform to the crowd. Therefore, more interactions are needed to eventually form opinion polarization.In fact, this paper focuses on the analysis of individual micro interactions. Through the model in this paper, we can accurately analyze the different performance of different preference individuals under the effect of heterogeneity revenue.

**Figure 27:**
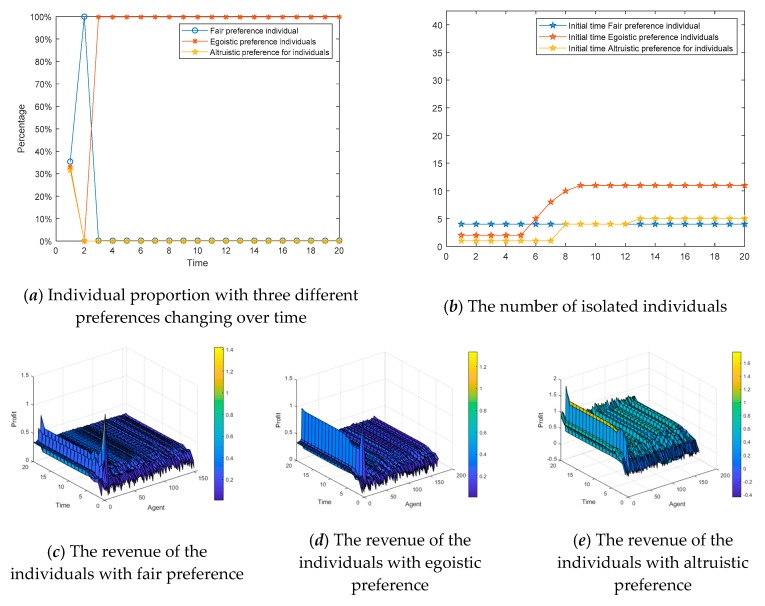
Specific preference analysis of a real case.

Subsequently, specific preference analysis of the real case is also discussed, as shown in Figure 27. Figure 27a describes the proportion of individuals with three different preferences in the network. Figure 27b shows the number of isolated individuals with different initial preferences. We assume that at the initial moment, each of these three preferences accounts for one-third. Figure 27c–e represents the interactive revenue of the individuals with three different preferences, respectively.

In Figure 27, at the initial moment, the individual preferences change greatly. Firstly, their preferences change to the fair preference, and then quickly change to the egoistic preference. From the perspective of isolated individuals, the number of individuals with an egoistic preference withdrawing from public opinion interactions is the largest. From the perspective of revenue, the individuals with an altruistic preference have the largest revenue in the network.

## 6. Conclusions

In this paper, social preference theory and revenue function are integrated into the model of public opinion polarization, and the influences of individuals with different preferences on public opinion polarization are also analyzed. The following conclusions can be made:

(1) Different social preferences held by individuals have different influences on public opinion polarization effects. Individuals with egoistic preference have fewer benefits through interaction in the network, so their interaction intention is low. The individuals with altruistic preference have the highest willingness to interact and the highest revenue, while ones with fair preference are in the middle.

(2) Through the simulation, we find that if there is only a single preference in the network, the degree of public opinion polarization is significantly lower than mixed preferences in the network. In addition, when the public opinion polarization phenomenon occurs, individuals will tend to interact with ones with different preferences.

(3) Generally, individuals’ preference will alter with the change of interaction revenue. However, through simulation, we can see that at the initial moment, most individuals’ preference will become an altruistic preference, but the period is very short. Then, individuals with an egoistic preference in the network will begin to increase and dominate, while ones with fair preferences account for the least.

However, the following limitations still need to be studied in the future.

(1) When constructing the network, only the node exit rule was considered, but the node increase rule accompanied by the hot event propagation at the initial time was not considered [37,38]. In addition, the connections of all individuals were treated as bidirectional ones when the network was constructed, while in reality there are many unidirectional connections of nodes. Therefore, the node increase rule and unidirectional network structure should be explored in the future.

(2) Through the simulation of the real cases, there exists certain relevance between the change of an individual’s social preference and the public opinion inversion phenomenon [39]. As a result, the relationships between them also need to be further discussed in future studies.

(3) There is still no theoretical basis for the variability of preferences held by individuals at different stages, and further research is needed on the underlying reasons for the change of preferences held by individuals.

## Figures and Tables

**Table 1:** Network parameters.

Node Number	Average Length	Clustering Coefficient	Average Degree
500	1.5602	0.45575	219.448
